# Demography of Symbiotic Nitrogen-Fixing Trees Explains Their Rarity and Successional Decline in Temperate Forests in the United States

**DOI:** 10.1371/journal.pone.0164522

**Published:** 2016-10-25

**Authors:** Wenying Liao, Duncan N. L. Menge

**Affiliations:** 1 Department of Ecology, Evolution and Environmental Biology, Columbia University, New York, New York, United States of America; 2 Department of Ecology and Evolutionary Biology, Princeton University, Princeton, New Jersey, United States of America; Chinese Academy of Forestry, CHINA

## Abstract

Symbiotic nitrogen (N) fixation is the major N input to many ecosystems. Although temperate forests are commonly N limited, symbiotic N-fixing trees (“N fixers”) are rare and decline in abundance as succession proceeds–a challenging paradox that remains unexplained. Understanding demographic processes that underlie N fixers’ rarity and successional decline would provide a proximate answer to the paradox. Do N fixers grow slower, die more frequently, or recruit less in temperate forests? We quantified demographic rates of N-fixing and non-fixing trees across succession using U.S. forest inventory data. We used an individual-based model to evaluate the relative contribution of each demographic process to community dynamics. Compared to non-fixers, N fixers had lower growth rates, higher mortality rates, and lower recruitment rates throughout succession. The mortality effect contributed more than the growth effect to N fixers’ successional decline. Canopy and understory N fixers experienced these demographic disadvantages, indicating that factors in addition to light limitation likely contribute to N fixers’ successional decline. We show that the rarity and successional decline of N-fixing trees in temperate forests is due more to their survival disadvantage than their growth disadvantage, and a recruitment disadvantage might also play a large role.

## Introduction

Nitrogen (N) limits primary production in many terrestrial ecosystems [1], and comes primarily from biological N fixation [2]. At local scales, symbiotic N fixation (SNF) can be an enormous N input, bringing over 100 kg N ha^-1^ yr^-1^ in to some ecosystems [3], but symbiotic N fixers are rare in many ecosystems [4]. Despite the crucial role of SNF in global and local biogeochemistry, there are major gaps in our understanding of the ecology of symbiotic N-fixing plants.

In particular, symbiotic N-fixing trees (hereafter, “N fixers”) play a major role in a challenging ecological paradox [4]. Most temperate forests experience N limitation throughout succession [5,6]. N limitation should favor N fixers, which can acquire N directly from the atmosphere. However, symbiotic N-fixing trees comprise <1% of tree basal area in the coterminous U.S. [7], and are also rare in other temperate forests. Furthermore, N fixers decrease in abundance as temperate forests age [7,8].

Why are N fixers rare in N-poor temperate forests, and why do they decline through succession despite the persistence of N limitation? Evolutionary constraints are unlikely to explain their rarity: there would be thousands of extant high-latitude N-fixing woody species if N fixation were widely adaptive in temperate and boreal forests [7,9]. Therefore, the most likely class of explanation is that some inherent ecological or physiological constraint makes N fixation a maladaptive strategy in temperate forests [9]. Many ecological constraints have been hypothesized. Compared with non-fixers, N fixers might have greater demand for nutrients, such as phosphorus or molybdenum [4], or for light because of the high energetic cost [10] of fixing N [11,12]. Alternatively, they might be preferentially grazed because of N-rich plant tissue [11,13].

Our understanding of these ecological mechanisms is still coarse. An analysis of forest inventory data suggested that temperate N-fixing tree species are more light-demanding than non-fixing species [7], but that analysis did not clarify how light demand related to N fixation costs because rates of N fixation were not quantified. The other mechanisms are less well understood. Temperate N fixers are grazed more heavily [14–16] or are more palatable [17] than non-fixers in some studies, but not in others [18], although most of these studies were on herbaceous plants, not trees. Phosphorus is related to N fixer growth and activity in some studies [18–21], but not others [22,23].

A missing piece of this puzzle is how temperate N fixers and non-fixers differ in demographic (i.e. vital) rates such as growth, mortality, and recruitment. Understanding demographic dynamics of N fixers vs. non-fixers would provide 1) a proximate answer to the observed latitudinal and successional patterns, and 2) insights into the differences in evolutionary strategies of the two functional groups. These vital rates are a more proximate control on abundance [24] than physiological processes, and are easier to quantify at large scales. Furthermore, physiological processes influence vital rates. For example, higher photosynthetic capacity and respiration rates, which are often associated with high foliar N content [25,26], are related to faster growth rates in some species [27]. Thus, understanding vital rates might provide insights into physiological mechanisms. The study of demographic tradeoffs has a long history in ecology, particularly in temperate forests [28,29]. For example, light-demanding pioneering species often show an r strategy (“colonization”), growing rapidly in high light and dispersing far, but dying easily once over-topped. On the contrary, shade-tolerant species often have a K strategy (“exploitation”), balancing low maximum growth rates and short dispersal with low mortality rates [29]. Given their shade intolerance [7], we expect N-fixing trees to be on the high-growth, high-mortality end of this tradeoff axis.

Here, we reveal which demographic mechanisms explain the low abundance and successional decline of N fixers in temperate forests. We used forest inventory data from the coterminous U.S.A. to address three questions: (1) How do N fixer growth, mortality, and recruitment rates compare with the corresponding non-fixer rates in temperate forests? (2) How do these differences in N fixer vs. non-fixer demographic rates change along succession? (3) What are the relative contributions of each demographic process to the low abundance and successional decline of N fixers in temperate forests? We examined these questions for the entire functional group of N fixers, for individual N-fixing species, and for trees in different canopy positions (canopy vs. understory). If shade intolerance is a dominant driver of low N fixer abundance, we would expect N fixers to have demographic advantages compared with non-fixers in high light, but disadvantages in shade.

## Materials and Methods

### Forest inventory data

We used version 5.1 of the U.S. Forest Service’s Forest Inventory and Analysis (FIA) database (http://www.fia.fs.fed.us/). The FIA is a systematic survey of forest plots (~ 1 plot per 2400 ha) across the coterminous U.S. Plot censuses are taken every 5–10 yr. Each plot has four subplots with 7.3 m radius for tree [diameter at breast height (dbh) ≥12.7 cm] measurements, and four subplots with 2.1 m radius for sapling (dbh 2.54–12.7 cm) measurements. Stand age is defined by FIA as “the average age of the live trees not overtopped in the predominant stand size-class” and estimated by coring several live trees [30]. FIA’s stand age variable is monotonically related to time since last stand-replacing disturbance [31]. Because of the way stand age is defined, we cannot distinguish between primary and secondary succession, although the majority of plots likely represent secondary succession.

We restricted our analysis to plots that (1) were not plantations, (2) were fully sampled, (3) had no evidence of harvesting or logging during each census period, (4) had more than one census (to allow for rate calculations), and (5) had at least one symbiotic N-fixing tree present. A total of 2639 plots met these criteria (S1 Fig), with 2–4 censuses each. Among all the plots, 2513 plots are in the eastern U.S. (longitude > -100°), and 126 plots are from the western U.S. (longitude ≤ -100°). We only examined plots with N fixers because these best represent competition between N fixers and non-fixers. Among 69,581 trees in our data set, 9488 of them are saplings.

### N-fixing tree taxa

Species were classified as N fixers or non-fixers by referencing published reports on nodulation or N fixation activity [32,33] and the GRIN database (http://www.are-grin.gov/~sbmljw/cgi-bin/taxnodul.pl), following Menge *et al*.[34]. In the coterminous U.S. FIA dataset, there are five rhizobial (*Olneya*, *Robinia*, *Acacia*, *Prosopis*, and *Albizia*) and three actinorhizal genera (*Alnus*, *Cercocarpus*, and *Elaeagnus*). Of the 11 species, *Robinia pseudoacacia* was the most abundant, followed by *Cercocarpus ledifolius* and *Alnus rubra*. For species-level estimates we only used taxa that were represented by at least 100 data points in our dataset.

### Demographic rate calculations

Because FIA tags and remeasures individual trees, we calculated growth and mortality on an individual basis, largely following Condit *et al*. [35]. Because we could not identify parents of new recruits, we calculated recruitment at the plot level. Individual tree relative growth rates *G* were calculated as logarithm of diameter increment $\frac{ln(dbh_{t+\Delta t})-ln(dbh_{t})}{\Delta t}$, where Δ*t* is the time between the two censuses. Following Condit *et al*.[35], we assumed that negative growth rates were small but undetectable positive growth. We therefore adjusted negative growth measurements to $\frac{\left(ln(dbh_{t}+\frac{1}{2}MDL))-ln(dbh_{t}\right)}{\Delta t}$, where the minimum detection limit (MDL) was 0.05 cm.

We calculated mortality for each census interval for each individual. For fits without a diameter effect on mortality, and for display purposes, we used these data to calculate the community-level mortality rate *m* as $\frac{ln(N_{t})-ln(S_{t+\Delta t})}{\Delta t}$, where *N*_*t*_ and *S*_*t+*Δ*t*_ denote the population size at one census and the number of those still alive at the next interval, respectively. Finally, the recruitment rate *λ* was calculated as $\frac{R_{t+\Delta t}}{N_{t}∙\Delta t}$, where *R*_*t+*Δ*t*_ denotes number of trees recruited in the time interval Δ*t*.

### Crown-class analysis

We categorized trees with FIA crown classes open grown, dominant, and co-dominant as canopy trees, and those with FIA crown classes intermediate and overtopped as understory trees [7].

### Statistics

We used maximum likelihood estimates to model each demographic rate as a function of stand age and fixing status, and for growth and mortality, as a function of individual tree size. We compared different non-linear functions of stand age. We assumed growth rates were lognormal [35], mortality rates were binomial [35], and recruitment rates were Poisson distributed [36].

We used mle2 [37] in R [38] to estimate maximum likelihood model parameters, and Akaike’s Information Criterion [36] to select the best fit model for each dataset. We used the population prediction interval method [36] to estimate confidence limits on these fits. The fits were plotted using the mean diameter of all trees within the functional group (i.e., plotted fits represent average-sized tree trends). Details of these analyses are in the Supporting Information (S1 Text).

### Individual-based model

To understand how successional dynamics of N-fixing vs. non-fixing trees depend on each demographic process, we used an individual-based simulation model, following Menge and Chazdon [34]. We initially ran the simulation using the maximum likelihood functions fit to each type (“all demographic effects”). To understand how each demographic process influenced the successional trajectory, we allowed demographic rates of N fixers and non-fixers to differ for the focal demographic process, while holding the other two the same. Details are in the Supporting Information (S2 Text).

We ran simulations for all N fixers and the three most abundant N fixer species. For *Cercocarpus* and *Alnus*, we had too few data to estimate recruitment, so instead of an “all demographic effect” scenario, we examined combined growth and mortality effects.

## Results

### Coterminous U.S.

**Fig 1:**
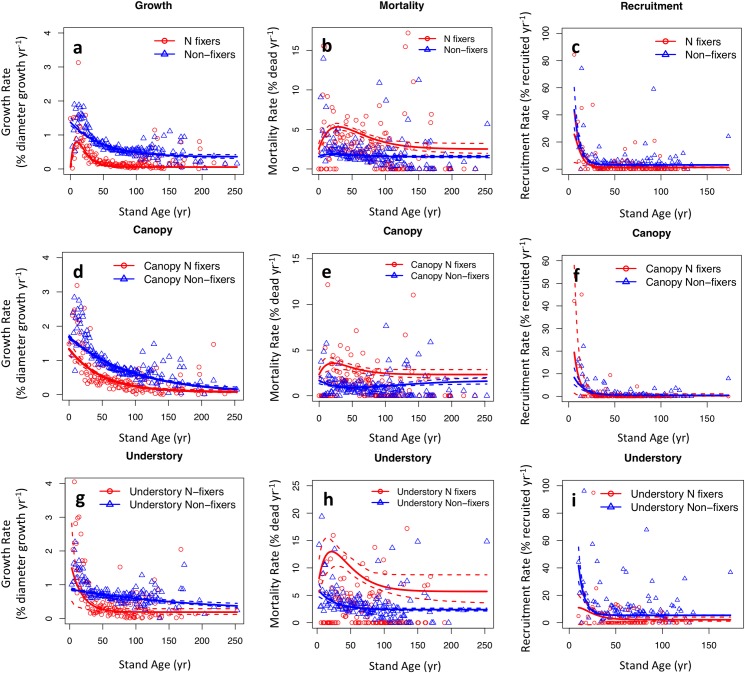
Demographic data and model fits for N-fixing vs. non-fixing trees in the coterminous U.S. Growth (a,d,g), mortality (b,e,h), and recruitment (c,f,i) are shown for all trees (a-c), canopy trees (d-f), and understory trees (g-i). Red circles (N fixers) and blue triangles (non-fixers) indicate geometric means of demographic rates at each stand age. Non-fixers are those that exist in the same plots as N fixers. Geometric means are shown for visual clarity, but models were fit to the entire dataset. Model fit means (solid lines) and 95% confidence intervals (dashed lines) are shown for average-sized trees for growth and mortality (see Supporting Information for details of statistics, S1 Text).

N fixers grew slower, died faster, and recruited less compared with non-fixers across the coterminous U.S. (Fig 1A, 1B and 1C). The growth rate of average-sized non-fixers decreased with forest age, whereas that of N fixers peaked before declining (Fig 1A). Throughout succession, N fixers had lower average growth rates than non-fixers. The curve appears not to be a perfect fit to the data because fits were plotted for average sized trees, but trees in younger forests were smaller than average. Mortality rates of both N fixers and non-fixers peaked around 20 yr, but N fixers died faster throughout succession, with the largest disadvantage coming in younger stands (Fig 1B). Recruitment rates of both N fixers and non-fixers decreased as the forest aged. Non-fixers appeared to recruit more than N fixers, although our confidence in this recruitment difference in early succession is low (confidence intervals overlap) (Fig 1C). Instead of showing a high growth with high mortality along the growth-mortality trade-off spectrum, our results indicate that N fixers experience demographic inferiorities in both growth and mortality.

**Fig 2:**
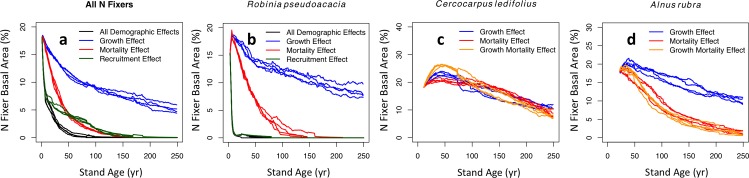
Simulation results from the individual-based model. Simulations use fits for all N fixers (a) and the three most common N-fixing tree species: *Robinia pseudoacacia* (b), *Cercocarpus ledifolius* (c), and *Alnus rubra* (d). Colors show different model scenarios. For all N fixers (a), four scenarios are shown: Differences in all three demographic processes (black), as determined by statistical fits shown in Fig 1A–1C, and differences in either growth rate (blue), mortality rate (red), or recruitment rate (green). Therefore, each colored curve shows how an N fixer effect on one demographic process contributes to the overall successional trend. Four simulations are shown for each scenario. Scenarios for *Robinia* (b) are the same as for all N fixers, except they use the fits in Fig 3A, 3B and 3C. For *Cercocarpus* (c, using fits in Fig 3D and 3E) and *Alnus* (d, using fits in Fig 3F and Vg), only growth (blue), mortality (red), and combined growth and mortality effects (orange) are shown.

Our individual-based model showed that differences between N fixers and non-fixers in all three demographic processes—growth, mortality, and recruitment—contributed to the successional decline of N fixers, but to varying degrees (Fig 2). The recruitment effect (i.e. N fixers recruit less than non-fixers throughout succession) had the largest potential effect, but given the uncertainty in the recruitment difference, we treat this result with caution. The mortality effect (i.e. N fixers die more frequently than non-fixers throughout succession) was stronger than the growth effect (i.e. N fixers grow slower than non-fixers throughout succession). If the mortality effect were the only difference between N fixers and non-fixers, it would cause N fixers to decrease from the starting value of these simulations to <5% of basal area by 50 yr. In contrast, the growth effect on its own takes more than 250 yr to bring N fixers below 5% of basal area. Although N fixers experience demographic disadvantages throughout succession, the population growth rate (*N*_*t+1*_*/N*_*t*_) calculated from our simulations remained greater than 1.

### Species-level analysis

**Fig 3:**
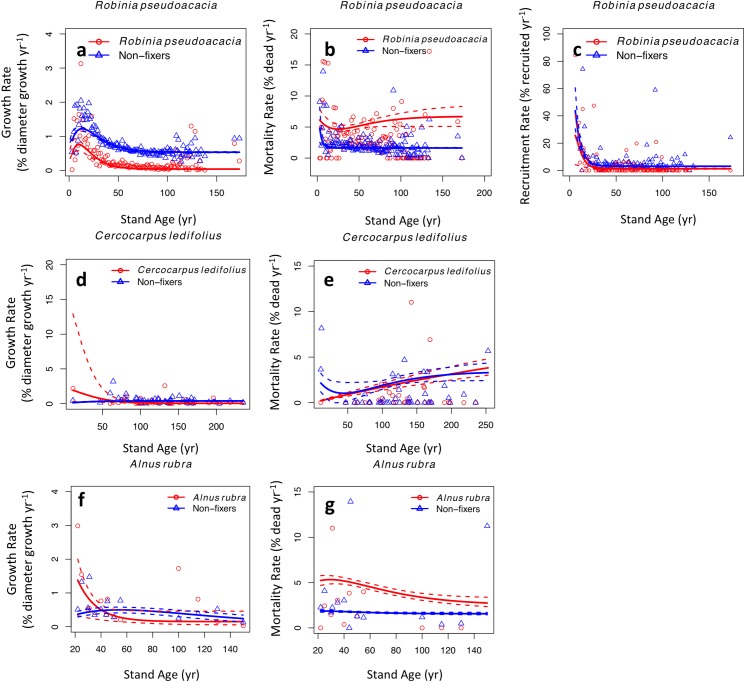
Demographic rates of the three most abundant N-fixing tree species compared with all non-fixing trees found in the same plots. Details are the same as Fig 1.

Demographic differences between *Robinia* and its co-occurring non-fixers (Fig 3A, 3B and 3C) were similar to differences between all U.S. N fixers and their co-occurring non-fixers (Fig 1A, 1B and 1C). *Cercocarpus* and *Alnus* tended to grow faster than non-fixers early in succession, but this trend decreased and tended toward a disadvantage as the forest aged (Fig 3D and 3F). *Cercocarpus* survived better in early succession, but otherwise died at a similar rate as its non-fixing neighbors (Fig 3E). *Alnus* died at a higher rate than co-occurring non-fixers throughout succession (Fig 3G).

The individual-based model showed that the mortality effect was much stronger than the growth effect for *Robinia* (Fig 2B) and *Alnus* (Fig 2D), similar to the overall N fixer results (Fig 2A). For *Cercocarpus* (Fig 2C), the growth effect was marginally stronger.

### Crown-class analysis

N-fixing trees were relatively more abundant in the canopy than in the understory (S2 Fig). Canopy non-fixing trees grew faster than canopy N-fixing trees throughout succession (Fig 1D). At the earliest stages of succession, understory N fixers tended to grow faster than understory non-fixers, but for the remainder of succession, non-fixers grew faster than N fixers (Fig 1G). Both understory and canopy N fixers died faster than their co-occurring non-fixers (Fig 1E and 1H). Similar to the recruitment trend of all trees (Fig 1C), understory non-fixers recruited faster than N fixers, especially in early ages (Fig 1I). Recruitment for canopy trees was similar throughout succession (Fig 1F).

## Discussion

Across the coterminous U.S., N fixers grew slower and died faster, and tended to recruit slower compared with neighboring non-fixers. These combined effects can explain the low abundance of N fixers and their successional decline. Our individual-based model indicated that mortality had a stronger effect than growth on the community trajectories of N fixers and non-fixers. At the species level, *Robinia pseudoacacia* and *Alnus rubra* were similar to all N fixers across the coterminous U.S., except that *Alnus* growth tended to be higher than non-fixers at the earliest stages of succession. In contrast, *Cercocarpus ledifolius* was demographically similar to its neighboring non-fixers.

In this section, we 1) explain potential mechanisms that result in the slow growth, high mortality, and low recruitment of N fixers; 2) explain why N fixers did not show clear growth-mortality trade-offs; 3) discuss the effect of physiological constraints, such as shade-tolerance, on demographic rates of N fixers; 4) discuss processes that maintain the presence of N fixers given their demographic inferiority; 5) conclude by comparing differences of N fixer demographics between temperate and tropical forest succession, and by identifying the key driver of this difference.

### What determines the demographic inferiority of N fixers?

All N fixers, *Robinia*, and *Alnus* beyond early succession grew slower than non-fixers throughout succession. This contradicts our expectation, given that N fixers are typically thought to be fast growers [34,39]. *Robinia* and *Alnus*, in particular, can grow rapidly, especially in their juvenile stages [40–42]. Why, then, did many N fixers grow slower in the FIA plots? Two explanations seem plausible. 1) The comparisons we examined here are to co-occurring non-fixers, not to “average” trees, and these co-occurring non-fixing species might also grow fast. 2) The realized growth in natural settings might be well below maximum growth potential due to various constraints. The potential constraints—light, water, nutrients, herbivores, and disease—affect all plants, but might affect N fixers more than non-fixers [4,11]. For instance, *Robinia* is severely damaged by insects and disease [43]. N itself might constrain N fixers relative to non-fixers, given anthropogenic changes to N availability over the past few decades. Much of the northeast U.S. receives on the order of 5–10 kg N ha^-1^ y^-1^ [44], which increases the growth of most non-fixing species in this region [5]. If N-fixing species continue to fix N even after N deposition has alleviated N limitation—i.e., if they are obligate or incompletely down-regulating N fixers [45,46]—then they would be wasting energy, which would put them at a growth disadvantage compared to non-fixers. There is evidence that *Alnus* is obligate [3,47], which could help explain its growth disadvantage, although N fixation strategies for *Robinia* and *Cercocarpus* are not as clear.

With the exception of *Cercocarpus*, N fixers died faster than non-fixers throughout succession. What could cause N fixers to suffer higher mortality than non-fixers? Insect and fungal infection, discussed above in relation to slow growth, could also be a major contributor to high mortality [48], and is in line with findings from other ecosystems [14–17] that N fixers suffer higher herbivory. High foliar N content in N fixers, which is associated with higher foliar respiration [25], could lead to greater carbon starvation, which has been posited to be lethal in periods of water stress [49]. On the contrary, high tissue N can allow similar photosynthetic rates at lower stomatal conductance, i.e., higher water use efficiency [50], as has been found in N-fixing legumes [51]. Other traits, such as low wood density in alder [41], which is associated with cavitation risk [52] and toppling [53], could also contribute to mortality, although *Robinia* has high wood density [54].

N fixers tended to recruit less well than non-fixers, although we could only assess recruitment for *Robinia*. Recruitment limitation has two key elements, dispersal limitation and establishment limitation (i.e. local competition suppresses the sprouting of invading species) [55]. *Robinia* has heavy seeds, which generally disperse slowly [56], so dispersal limitation seems plausible.

### Why is there no clear growth-mortality trade-offs for N fixers?

Given their shade-intolerance and specialization in early successional forests in temperate regions, we expected N fixers to have high growth with high mortality along the growth-mortality trade-off spectrum. However, our results indicate demographic inferiorities of N fixers in both growth and mortality. This seemingly surprising result is likely explained by several reasons [57]: 1) The growth-mortality trade-off concerns growth rate in optimal conditions versus mortality in challenging conditions [29,58]. Our observations spanned a wide range of conditions, so did not specifically target the conditions under which the growth/survival tradeoff should emerge. We did stratify results by canopy position (Fig 1D–1I), which does show growth in the canopy (presumably higher light) and mortality in the understory (presumably low light). This stratification did not bring out a growth/mortality trade-off, but other environmental conditions are not accounted for by canopy position. 2) The growth-mortality trade-off is strongest in seedlings and saplings, but weak in adult trees [58]. Our analysis did not include seedlings, and only a small proportion of trees (<14%) were saplings.

### How do N fixers’ demographic rates inform our understanding of their physiological constraints?

Our results shed light on the relative roles of previously proposed physiological constraints on N fixers. Previous research with FIA data suggested that N fixers are less shade tolerant than non-fixers [7], consistent with our finding that N fixers tend to be proportionally more abundant in the canopy than in the understory (S2B Fig). Despite the important role of shade intolerance of N fixers, our findings suggest that factors other than shade intolerance are also important. Specifically, the fact that N fixers grew slower and died faster in the canopy as well as the understory strongly suggests that N fixers’ disadvantage is not driven entirely by shade intolerance. Therefore, other constraints, such as greater herbivory or demand for other nutrients [4,11,13], likely play important roles.

### Why are N fixers present?

Given that growth, survival, and recruitment rates were all worse for N fixers than non-fixers, why are N fixers present? There are several possible explanations. 1) There are only a few very young FIA plots that met all of our required conditions. The demographic trends in early succession from our analysis might be a misrepresentation because of a small sample size. It is plausible that N fixers have demographic advantages very early in succession, which ensures their initial establishment. 2) Whereas we showed demographic disadvantages of N fixer saplings and trees, dynamics of seedlings might be different. Survival and longevity of N fixer seedlings might be higher than those of non-fixers because of high parental investment (eg. high seed N content [59]). 3) Although on average N fixers had demographic disadvantages compared with non-fixers along succession, N fixers might also show high variation in demographic rates. In favorable conditions with sufficient light, water, and nutrients other than N, N fixers might be strong competitors. This variation would allow some N-fixing individuals to perform well, ensuring their persistence in temperate forest. 4) Our simulation results show that N fixers have positive population growth rate (*N*_*t+1*_*/N*_*t*_ >1), suggesting that even though they experience demographic disadvantages compared to non-fixers, N fixers can persist. 5) N fixers’ current distribution might be a legacy of a time when atmospheric N deposition was lower. If their current demographic disadvantages stem from higher N deposition, and higher N deposition continues or increases, then we might expect them to become even rarer in the decades to come.

### How do demographic dynamics differ in temperate vs. tropical forest succession?

Whereas N-fixing trees in the coterminous U.S. grew slower and survived worse than their co-occurring non-fixers, N-fixing trees in Northeastern Costa Rica grew faster and survived better than non-fixing trees, particularly early in succession [34]. N-fixing trees in the canal region of Panama also grew faster than non-fixers early in succession, and had higher net recruitment (recruitment compared to mortality) [39]. In Costa Rica, greater survival was N fixers’ key to success [34], just as lower survival was the key to N fixers’ decline in our results. Tropical and temperate N-fixing trees differ in a number of interesting ways; together, these recent results suggest that N fixer vs. non-fixer survival is a key driver of these differences.

## Supporting Information

S1 Fig Distribution of plots where N fixers and non-fixers co-occur. (DOCX)

S2 Fig Fraction of N fixers and non-fixers in canopy and understory class. (DOCX)

S1 Table Akaike’s Information Criteria for Models. (DOCX)

S1 Text Details of Statistics. (DOCX)

S2 Text Details of Individual-based Model. (DOCX)
