# Supplementary material for: Demography of Symbiotic Nitrogen-Fixing Trees Explains Their Rarity and Successional Decline in Temperate Forests in the United States
Source: PLoS One. 2016 Oct 25;11(10):e0164522. doi: 10.1371/journal.pone.0164522 (PMC5079550; doi:10.1371/journal.pone.0164522)
Supplement: S1 Fig — (DOCX) [file pone.0164522.s001.docx]

***Liao et al. Demography of Symbiotic N-fixing Trees***

**S1 Fig.** Distribution of the 2639 FIA plots with N fixers co-occuring with non-fixers that met our selection criteria. See main text for selection criteria. Colors correspond to the number of plots in each 1° latitude x 1° longitude grid cell. White space indicates that no plots in the grid cell met the criteria. Note the logarithmic color scale.
