# Supplementary material for: Demography of Symbiotic Nitrogen-Fixing Trees Explains Their Rarity and Successional Decline in Temperate Forests in the United States
Source: PLoS One. 2016 Oct 25;11(10):e0164522. doi: 10.1371/journal.pone.0164522 (PMC5079550; doi:10.1371/journal.pone.0164522)
Supplement: S2 Fig — (DOCX) [file pone.0164522.s002.docx]

***Liao et al. Demography of Symbiotic N-fixing Trees***

**S2 Fig.** Prevalence of N fixers in different canopy positions in FIA plots that met our selection criteria. (a) Numbers and (b) fractions of N fixers (red) and non-fixers (blue) are shown in the canopy and the understory.
