# Supplementary material for: Demography of Symbiotic Nitrogen-Fixing Trees Explains Their Rarity and Successional Decline in Temperate Forests in the United States
Source: PLoS One. 2016 Oct 25;11(10):e0164522. doi: 10.1371/journal.pone.0164522 (PMC5079550; doi:10.1371/journal.pone.0164522)
Supplement: S1 Table — (DOCX) [file pone.0164522.s003.docx]

***Liao et al. Demography of Symbiotic N-fixing Trees***

**S1 Table.** Summary of Akaike’s Information Criteria for all models with diameter effect fitted in our analysis.

|  | N Fixers | | |
| --- | --- | --- | --- |
|  | Growth | Mortality | Recruitment |
| All | Saturating 558.5  Sigmoid 183.5  Ricker 0 | Saturating 2.6  Ricker 0 | Saturating^1^ 42.2  Saturating^2^ 0.3  Ricker^1^ 11.4  Ricker^2^ 0 |
| *Robinia pseudoacacia* | Saturating 3.2  Sigmoid 3.1  Ricker 0 | Saturating 6.7  Ricker 0 | Saturating^1^ 9.8  Saturating^2^ 0.3  Ricker^1^ 10.4  Ricker^2^ 0 |
| *Cercocarpus ledifolius* | Saturating 3.4  Sigmoid 0  Ricker 17 | Saturating 16.9  Ricker 0 |  |
| *Alnus rubra* | Saturating 0  Sigmoid 2.2  Ricker 0.2 | Saturating 42.2  Ricker 0 |  |

|  | Non-fixers | | |
| --- | --- | --- | --- |
|  | Growth | Mortality | Recruitment |
| All | Saturating 0  Sigmoid 8.8  Ricker 11.6 | Saturating 2.6  Ricker 0 | Saturating^1^ 0.1  Saturating^2^ 0  Ricker^1^ 215.1  Ricker^2^ 209.4 |
| *Robinia pseudoacacia* | Saturating 55.9  Sigmoid 26.5  Ricker 0 | Saturating 4.2  Ricker 0 | Saturating^1^ 1.0  Saturating^2^ 0  Ricker^1^ 329.5  Ricker^2^ 321.7 |
| *Cercocarpus ledifolius* | Saturating 0  Sigmoid 42.2  Ricker 40.2 | Saturating 6.9  Ricker 0 |  |
| *Alnus rubra* | Saturating 1.7  Sigmoid 6.5  Ricker 0 | Saturating 2.7  Ricker 0 |  |

* For recruitment analysis, shape function^1^ considered fecundity effect of trees within same functional type. Shape function ^2^ considered the competitive effect of all trees, regardless of functional types. (See **S1 Text** for details.)
