# Supplementary material for: Demography of Symbiotic Nitrogen-Fixing Trees Explains Their Rarity and Successional Decline in Temperate Forests in the United States
Source: PLoS One. 2016 Oct 25;11(10):e0164522. doi: 10.1371/journal.pone.0164522 (PMC5079550; doi:10.1371/journal.pone.0164522)
Supplement: S1 Text — (DOCX) [file pone.0164522.s004.docx]

***Liao et al. Demography of Symbiotic N-fixing Trees***

**S1 Text. Statistics**

Our maximum likelihood estimates of demographic rates were largely adapted from the approach of Condit *et al*. (2006).

*Growth*

We calculated growth rates of tree *k* at census interval Δ*t* as:

*G_k_*,*_t_* = (ln(*D_k,t_*_+Δ_*_t_*) – ln(*D_k,t_*))/Δ*t.*

We excluded outliers that were below the 1^st^ percentile or above the 99^th^ percentile of growth. The resulting data were heavily right-skewed and contained some negative growth. Assuming that negative values of growth are a combination of either measurement error or non-growth process such as shrinkage due to low water content, we corrected negative growth following the approach of Condit *et al*. (2006):

Negative growth values were set to *G_k,t_* = (ln(*D_k,t_* + ½*MDL) – ln(*D_k,t_***))/**Δ*t****,*** with MDL (minimum detection limit) as minimum recordable growth increment (0.05 cm). The corrected data appeared lognormal.

We then used a maximum likelihood model of growth as a function (*φ*) of successional age (*A*), tree diameter (*D*), and whether or not the individual is a potential N fixer (*F*):

*G_k,t_ = φ*(*A_k,t_*,*D_k,t_*,*F_k_*)exp(*ε_k,t_*),

ln(*G_k,t_*) = ln(*φ*(*A_k,t_*,*D_k,t_*,*F_k_*) + *ε_k,t_*

where *ε_k,t_* is the error term, distributed as *P*(*ε_k,t_*) (which we assume to be normal with mean 0 and variance *s*^2^), so

*ε_k,t_* = ln(*G_k,t_*) *–* ln(*φ*(*A_k,t_*,*D_k,t_*,*F_k_*))

The general negative log likelihood function is

$$NLL=-\sum\left\{ \begin{aligned} \ln\left( \int_{-\infty}^{{MDL(D}_{k,t})} Normal\left( \ln\left( G \right)|\ln\left( \varphi_{k,t}\left( A_{k,t}D_{k,t}F_{k} \right) \right),s \right)d ln(G) \right), G_{k,t} \leq0 \\ \ln\left( Normal\left( {ln(G}_{k,t})|\ln\left( \varphi_{k,t}\left( A_{k,t}D_{k,t}F_{k} \right) \right),s \right) \right) , G_{k,t}>0 \end{aligned} \right.$$

We compared multiple functions against each other: The function *φ*, which shows the expected relative growth rate for a given stand age, tree diameter, and functional type (N fixer versus non-fixer), was:

${(\varphi}_{k,t})_{1}=\left( a_{k}-\left( b_{k}-a_{k} \right)*exp\left( \frac{-A_{k,t}}{c_{k}} \right) \right){(\frac{D_{k,t}}{\hat{D}})}^{d_{k}}$ (Saturating effect of age)

${(\varphi}_{k,t})_{2}=(a_{k}+\frac{b_{k}-a_{k}}{1+exp(-k_{k}(A_{k,t}-c_{k}))}){(\frac{D_{k,t}}{\hat{D}})}^{d_{k}}$ (Sigmoid effect of age)

${(\varphi}_{k,t})_{3}=(a_{k}+c_{k}\left( k_{k}-a_{k} \right)A_{k,t}*exp(-c_{k}A_{k,t})) {(\frac{D_{k,t}}{\hat{D}})}^{d_{k}}$ (Right-skewed effect of age)

We then used the function mle2 in the bblme package (Bolker & R Core Team, 2014) to calculate the maximum likelihood estimates of each parameter and their standard deviations.

*Mortality*

Following most of the framework in Condit *et al*. 2006, annual mortality rate was calculated from the population dynamics term *dN/dt* = –*mN*; *m* = [ln(*N*(*t*)) – ln(*N*(*t*+Δ*t*))]*/*Δ*t*. We defined *N_j,i,t_* as the number of individuals in plot *j* of type *i* (N fixer or non-fixer, indexed by *f*) at census *t*, and *S_j,i,t_* as the number of individuals that have survived to the next census. Now, *m_j,i,t_* = [ln(*N_j,i,t_*) – ln(*S_j,i,t_*)]*/*Δ*t*.

Similar to *φ* above, we assumed that *m_k,t_* was a function of forest age *A*, tree diameter *D*, and functional type *F* associated with the individual *k*.

We looked at cases assuming diameter does or does not have an effect. Then we fit our data to different shape functions, similar to the growth rate analysis. The following equations show shape functions with no diameter effect:

(*m _A(k,t)_*)_1_ = *a_k_* + (*b_k_* – *a_k_*)*exp(-*A_k,t_*/*c_k_*) (Saturating effect of age)

${(m}_{A(k,t)})_{2}=a_{k}+c_{k}\left( k_{k}-a_{k} \right)A_{k,t}*exp(-c_{k}A_{k,t})$ (Right-skewed effect of age)

Survival probability is given by $\pi_{k,t}=e^{-m_{k,t}\cdot\Delta t}$. To analyze these data, we assumed that the survival probability $\pi$ for an individual in a census is given by a special case of binomial distribution - bernoulli distribution, Bern(*S*|*N*, *π*), where *π* is the survival probability parameter (corresponding to data *m_k,t_* from above), N is 1, and S is 0 if the individual died or 1 if survived during the census inverval. Our negative log likelihood function was

$NLL=-\sum_{k,t} ln(Bern\left( S|N,\pi_{k,t} \right))$.

We also compared a version of each of these with a diameter effect where we multiplied the entire right hand side by $({\frac{D_{k,t}}{\hat{D}})}^{d_{k}}$, where $\hat{D}$ is the geometric mean tree diameter across all individuals.

*Recruitment*

We calculated recruitment as the number of new individuals in each plot during each census, and presented these data as a percent of the individual of that type (N fixer or non-fixer, indexed by *f*) present in the previous census. We analyzed recruitment data as Poisson-distributed, with the expected number coming into each plot being $\lambda_{j,t,f}*N_{j,t,f}$, where $N_{j,t,f}$ is the number of individuals of that type (N fixer or non-fixer) in that plot-year combination. Written thus, $\lambda_{j,t,f}*100$ is the recruitment rate in percent per year (as shown on our figures). Our negative log likelihood function, therefore, was

$NLL=-\sum_{j,t,f} \ln\left( Poisson\left( R_{j,t,f}|\lambda_{j,t,f}N_{j,t,f} \right) \right).$

As above, we allowed $\lambda_{j,t,f}$ to vary as a function of forest age, and also examined cases where it varied as a function of the size of trees in its plot. Similar to mortality, the approximate shape was not entirely clear, so we compared a variety of reasonable functions using AIC.

*λ_j,t,f_* (*A_j,t_*)_1_ = *a_j,t,f_* + (*b_j,t,f_* – *a_j,t,f_*)*exp(-*A_j,t_*/*c_j,t,f_*) (Saturating effect of age)

*λ_j,t,f_* (*A_j,t_*)_2_ = *a_j,t,f_* + *c_j,t,f_(k_j,t,f_-a_j,t,f_)*A_j,t_**exp(-*c_j,t,f_A_j,t_*) (Right-skewed effect of age)

Similar to mortality, we also examined cases for each *λ_j,t,f_* (*A_j,t_*) where tree size has an effect. In this case there was no one to one correspondence between a newly recruited individual as an individual in the previous census, so the relevant size was the geometric mean diameter of individuals within a plot. We envisioned two possible effects here: one was that some individuals (presumably larger) will have greater fecundity, in which case only the size of individuals of the same type matters; alternatively, this may show up as a competitive effect, where plots with more individuals have a lower chance of seeds germinating and surviving to next census. We therefore examined both cases, multiplying each of the *λ_j,t,f_* (*A_j,t_*) functions by two versions of $({\frac{D_{j,t,f}}{\hat{D}})}^{d_{j,t,f}}$, one where *D_j,t,f_*  is the geometric mean of all individuals of this type (N fixer or non-fixer) in this plot in the previous census interval, and another where *D_j,t_* is the geometric mean of all individuals in this plot in the previous census interval, regardless of type. In both cases, $\hat{D}$ was the geometric mean tree diameter across all individuals, regardless of plot, type, or year.
