# Supplementary material for: Demography of Symbiotic Nitrogen-Fixing Trees Explains Their Rarity and Successional Decline in Temperate Forests in the United States
Source: PLoS One. 2016 Oct 25;11(10):e0164522. doi: 10.1371/journal.pone.0164522 (PMC5079550; doi:10.1371/journal.pone.0164522)
Supplement: S2 Text — (DOCX) [file pone.0164522.s005.docx]

***Liao et al. Demography of Symbiotic N-fixing Trees***

**S2 Text. Individual-based Model**

We compiled N fixer and non-fixer diameter data from forest sites <10 yr as the initial starting population for our individual-based model. Based on the statistical functions described in Methods S1, growth rates, mortality rates, and numbers of recruits were calculated. Our model tracks the diameters of each individual tree, which were then used to calculate basal area percent for each stand age.

Each year, each individual has its diameter growth rate estimated by the growth function *G_k,t_ = φ*(*A_k,t_*,*D_k,t_*,*F_k_*). The individual survived each year based on a stochastic Bernoulli process with survival probability $\pi_{k,t}=e^{-m_{k,t}\cdot t}$, where *m* was estimated from the mortality functions described in Methods S1. The number of recruits was calculated by the population size at each time step multiplied by the recruitment rate, as estimated by the recruitment function *λ_j,t,f_* (*A_j,t_*). The simulation ran until 250 yr, which was the observed oldest forest age with fixer present in our data set.

To increase computational efficiency, when the number of live trees exceeded 5,000, we subsampled trees from this original population. We reduced the new population size to 10% of the original, but preserved a similar tree diameter distribution.
